# Physiological responses and proteomic changes reveal insights into *Stylosanthes* response to manganese toxicity

**DOI:** 10.1186/s12870-019-1822-y

**Published:** 2019-05-22

**Authors:** Pandao Liu, Rui Huang, Xuan Hu, Yidan Jia, Jifu Li, Jiajia Luo, Qin Liu, Lijuan Luo, Guodao Liu, Zhijian Chen

**Affiliations:** 1 Institute of Tropical Crop Genetic Resources, Chinese Academy of Tropical Agricultural Sciences, Haikou, 571101 China; 2 Institute of Tropical Agriculture and Forestry, Hainan University, Haikou, 570110 China

**Keywords:** Manganese toxicity, Oxidative stress, Antioxidant protection, Proteomics, *Stylosanthes*

## Abstract

**Background:**

Manganese (Mn), an essential element for plants, can be toxic when present in excess. Stylo (*Stylosanthes*) is a pioneer tropical legume with great potential for Mn tolerance, but its Mn tolerance mechanisms remain poorly understood.

**Results:**

In this study, variations in Mn tolerance were observed among nine stylo genotypes. Stylo genotype ‘RY5’ exhibited the highest Mn tolerance compared to the other tested genotypes, whereas ‘TF2001’ was a Mn-sensitive genotype. The mechanisms underlying the response of stylo to Mn toxicity were further investigated using these two genotypes with contrasting Mn tolerance. Results showed that stylo genotype RY5 exhibited Mn tolerance superior to that of genotype TF2001, showing lower reductions in leaf chlorophyll concentration, chlorophyll fluorescence parameters, photosynthetic indexes and plant dry weight under Mn toxicity. A label-free quantitative proteomic analysis was conducted to investigate the protein profiles in the leaves and roots of RY5 in response to Mn toxicity. A total of 356 differentially expressed proteins (DEPs) were identified, including 206 proteins from leaves and 150 proteins from roots, which consisted of 71 upregulated, 62 downregulated, 127 strongly induced and 96 completely suppressed proteins. These DEPs were mainly involved in defense response, photosynthesis, carbon fixation, metabolism, cell wall modulation and signaling. The qRT-PCR analysis verified that 10 out of 12 corresponding gene transcription patterns correlated with their encoding proteins after Mn exposure. Finally, a schematic was constructed to reveal insights into the molecular processes in the leaves and roots of stylo in response to Mn toxicity.

**Conclusions:**

These findings suggest that stylo plants may cope with Mn toxicity by enhancing their defense response and phenylpropanoid pathways, adjusting photosynthesis and metabolic processes, and modulating protein synthesis and turnover. This study provides a platform for the future study of Mn tolerance mechanisms in stylo and may lead to a better understanding of the potential mechanisms underlying tropical legume adaptation to Mn toxicity.

**Electronic supplementary material:**

The online version of this article (10.1186/s12870-019-1822-y) contains supplementary material, which is available to authorized users.

## Background

Manganese (Mn) is the second most abundant transition metal in the Earth’s crust and is widely distributed in soils, sediments and water as well as in biological materials. Mn exists in many forms, such as free metal ions and soluble or insoluble metal compounds [1]. The available Mn in soils ranges from 450 to 4000 mg kg^− 1^ and can easily increase with decreasing soil pH or under reducing soil conditions [2]. Although Mn in trace amounts is essential for humans, animals and plants, Mn is considered a heavy metal; at excessive levels in farmlands, it not only decreases crop productivity and quality but also threatens human health [3]. In humans, the accumulation of Mn affects the central nervous system, leading to the development of Parkinson-like disorders [4]. In plants, excess Mn toxicity causes adverse impacts at various morphological levels, leading to symptoms such as chlorosis and necrosis, crinkled leaves and brown spots and, ultimately, growth inhibition [5, 6].

Several toxic effects of Mn on plants at the physiological level have been reported, such as generating oxidative stress through the accumulation of reactive oxygen species (ROS) [7, 8], impairing leaf structure and chlorophyll biosynthesis, impeding photosynthesis and respiration [9], inhibiting the activities of several key enzymes and disturbing the absorption and translocation of other mineral elements [10]. For example, overproduction of ROS, including hydrogen peroxide (H_2_O_2_), superoxide anion (O_2_^−^), hydroxyl radical (·OH) and singlet oxygen (^1^O_2_), is one of the main effects of Mn toxicity, causing lipid peroxidation if these ROS are not adequately scavenged [11]. Furthermore, the net photosynthetic rate (Pn), maximum quantum yield of photosystem II (PSII) (*F*v/*F*m) and effective quantum yield of PSII (ΦPSII) of plants are significantly inhibited by Mn toxicity [12, 13].

To avoid the toxic effects of Mn exposure, plants have developed diverse Mn tolerance mechanisms associated with changes in molecular, biochemical and cellular processes [5, 14]. Increasing evidence shows that plants typically tolerate excess Mn through activation of the antioxidant system [11], sequestration of Mn into inactive subcellular sites [15] and generation of Mn chelates with protein-based, organic and inorganic complexes [16]. Important roles of antioxidant systems in response to Mn toxicity, including antioxidant enzymes and nonenzymatic components, have been suggested in many plants, such as common bean (*Phaseolus vulgaris*) [7], cowpea (*Vigna unguiculata*) [17], perennial ryegrass (*Lolium perenne*) [13] and wheat (*Triticum polonicum*) [11]. It has been demonstrated that the compartmentalization of Mn in apoplasts, vacuoles, Golgi and cell walls plays an important role in Mn tolerance and homeostasis [17–19]. For example, loss-of-function analyses of metal tolerance protein (MTP) demonstrate the importance of Mn sequestration into vacuoles in Mn tolerance in rice (*Oryza sativa*) and Arabidopsis [20, 21]. Recently, it has been shown that sequestration of Mn into Golgi-associated compartments by the function of *OsMTP11* is also important for Mn homeostasis in rice [22]. Additionally, organic acids exuded from the root apex can chelate Mn, thereby alleviating Mn toxicity via decreasing Mn uptake by the roots [23–25]. It is generally assumed that Mn-toxicity tolerance varies greatly with plant species, variety or genotype. Therefore, dissecting the mechanisms underlying the plant response to Mn toxicity can provide valuable information for improving crop cultivars by increasing their adaptation to Mn toxicity.

Stylo (*Stylosanthes* spp.) is a dominant legume used for livestock nutrition and soil improvement, especially in tropical and subtropical areas where acid soils are widely distributed [26]. It has been reported that *S. guianensis* exhibits higher Mn tolerance than many other legumes, such as *Medicago sativa*, *Trifolium repens*, *Leucaena leucocephala*, *Glycine javanica* and *Phaseolus atropurpureus* [27]. Recently, a higher Mn toxicity threshold was observed in stylo than in other reported legumes under Mn toxicity [25]. Furthermore, stylo has aluminum (Al) toxicity tolerance comparable to that of Al-tolerant rice [28, 29]. Accordingly, stylo has been recognized as a pioneer tropical legume with great potential for metal tolerance. Although great efforts have been made to identify the physiological and molecular mechanisms of Mn tolerance in stylo, they remain poorly understood, which can be attributed to the lack of genome information and limited resources, such as stylo mutants. Furthermore, few studies have integrated analyses of the physiological responses and proteomic profiles of both leaves and roots of plants under Mn toxicity. These studies have the potential to provide insights into plant responses to Mn toxicity. In this study, the effects of Mn toxicity on the growth performance of nine stylo genotypes were investigated. Subsequently, physiological changes in two stylo genotypes contrasting in Mn tolerance were further analyzed. Differentially protein profiles in the leaves and roots of the Mn-tolerant stylo genotype under Mn toxicity were explored using a label-free quantitative proteomics approach. The potential mechanisms underlying the response of stylo to Mn toxicity were considered.

## Results

### Variability of Mn tolerance in stylo

In this study, growth performance was first examined among nine *S. guianensis* genotypes subjected to excess Mn. Stylo growth was obviously affected by Mn toxicity but showed variation among different genotypes (Additional file 1). RY5 exhibited highest Mn tolerance compared to the other tested stylo genotypes, as reflected higher SPAD values and plant dry weight under Mn toxicity, whereas TF2001 is a Mn-sensitive genotype (Additional file 1). Subsequently, two stylo genotypes, RY5 and TF2001, contrasting in Mn tolerance, were further used to investigate the response of stylo to Mn toxicity. The results showed that SPAD values in the leaves of RY5 and TF2001 were decreased by 19.7 and 48.9% in the excess Mn treatment compared to their respective controls (Table 1). The SPAD values in RY5 were higher than those in TF2001 under Mn toxicity (Table 1). Chlorophyll fluorescence parameters were monitored to evaluate the photosynthetic performance. The *F*v/*F*m, ΦPSII, excitation pressure of PSII (1-qL) and electron transport rate (ETR) calculated from ΦPSII were declined in the two tested stylo genotypes under excess Mn conditions compared to their respective controls (Table 1). Furthermore, the Pn, intercellular CO_2_ concentration (Ci) and stomatal conductance (Gs) were decreased in stylo treated with excess Mn (Table 1). Interestingly, the chlorophyll fluorescence parameters and photosynthetic indexes in RY5 were higher than in TF2001 under excess Mn conditions (Table 1).

**Table 1:** Chlorophyll fluorescence parameters and photosynthetic indexes of two stylo genotypes under excess Mn treatment

Genotypes	Mn (μM)	SPAD	*F*v/*F*m	ΦPSII	1-qL	ETR	Pn (μmol m^− 2^ s^− 1^)	Ci (μmol mol^− 1^)	Gs (mol m^− 2^ s^− 1^)
RY5	5	35.6 ± 1.4a	0.82 ± 0.01a	0.498 ± 0.048a	0.618 ± 0.023a	209.2 ± 20.2a	31.7 ± 2.0a	132.8 ± 7.5a	0.334 ± 0.013a
	400	28.6 ± 2.2b	0.66 ± 0.04b	0.358 ± 0.045b	0.486 ± 0.032b	150.4 ± 19.0b	24.6 ± 1.5b	114.9 ± 2.5b	0.245 ± 0.013b
TF2001	5	34.8 ± 1.7a	0.82 ± 0.01a	0.476 ± 0.050a	0.622 ± 0.044a	199.9 ± 21.0a	32.1 ± 2.2a	130.9 ± 10.6a	0.341 ± 0.048a
	400	17.8 ± 1.5c	0.42 ± 0.07c	0.222 ± 0.014c	0.338 ± 0.014c	93.2 ± 5.8c	15.4 ± 3.6c	104.5 ± 3.4b	0.161 ± 0.009c

Four chlorophyll fluorescence parameters, including *F*v/*F*m, ΦPSII, 1-qL and ETR, and three photosynthetic indexes, including Pn, Ci and Gs were measured in stylo after 10 d of 5 or 400 μM MnSO_4_ treatments. Data are means ± standard errors (*n* = 4). The same letter represents no significant difference at the *P* = 0.05 level

**Fig. 1:**
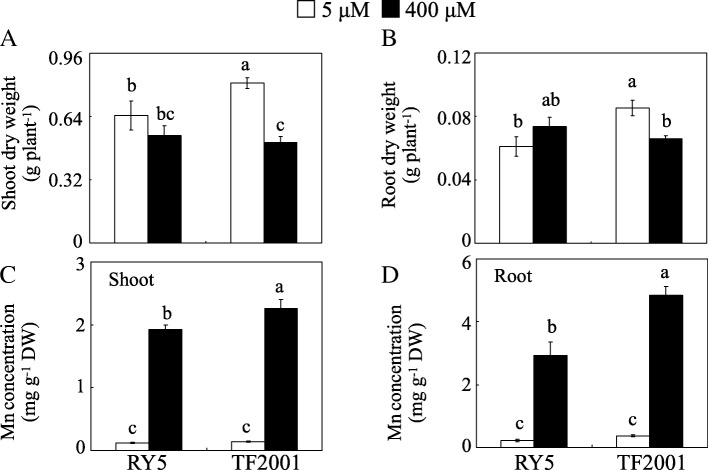
Effects of the different Mn treatments on stylo growth. (**a**) Shoot dry weight. (**b**) Root dry weight. (**c**) Shoot Mn concentration. (**d**) Root Mn concentration. After 30 d of normal growth, stylo seedlings were treated with 5 or 400 μM MnSO_4_ for 10 d. Each bar indicates the mean of four biological replicates with standard error. The same letter represents no significant difference at the *P* = 0.05 level

Excess Mn resulted in decreased shoot and root growth of TF2001 but not RY5. The shoot and root dry weights of TF2001 were 37.1 and 22.5% lower under Mn toxicity than those of the controls, respectively (Fig. 1a and b). Significant increases in Mn concentration were observed in both the shoots and roots of the two tested stylo genotypes in the excess Mn treatment relative to the control treatment. However, under excess Mn conditions, the Mn concentrations in shoots and roots were higher in TF2001 than in RY5 by 17.7 and 56.6%, respectively (Fig. 1c and d). These results suggest that RY5 is more Mn-tolerant than TF2001.

### Changes in H_2_O_2_ and malondialdehyde (MDA) levels in response to Mn toxicity

**Fig. 2:**
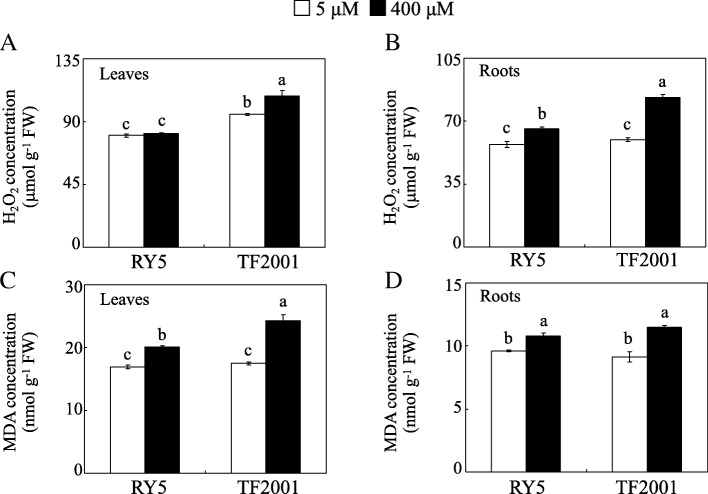
H_2_O_2_ and MDA concentrations in stylo in the two Mn treatments. H_2_O_2_ concentrations in leaves (**a**) and roots (**b**). MDA concentrations in leaves (**c**) and roots (**d**). After 30 d of normal growth, stylo seedlings were treated with 5 or 400 μM MnSO_4_ for 10 d. Each bar indicates the mean of four biological replicates with standard error. The same letter represents no significant difference at the *P* = 0.05 level

The H_2_O_2_ and MDA concentrations in stylo were examined at two Mn levels. The results showed that the H_2_O_2_ concentrations in the leaves and roots of TF2001 were increased by 13.6 and 39.3%, respectively, under excess Mn treatment, while H_2_O_2_ concentrations were only increased by 15.1% in the roots of RY5 when plants were exposed to Mn toxicity compared to the controls (Fig. 2a and b). Similarly, the MDA concentrations in the two tested stylo genotypes were significantly increased by excess Mn treatment (Fig. 2c and d). MDA concentrations in the leaves and roots of RY5 and TF2001 were 12.3–18.8% and 25.6–39.0% increased under excess Mn treatment, respectively, compared to the controls (Fig. 2c and d). Although increases in the concentrations of H_2_O_2_ and MDA were observed in both stylo genotypes subjected to Mn toxicity, RY5 maintained lower levels of H_2_O_2_ and MDA than TF2001 under Mn toxicity (Fig. 2).

### Response of the antioxidant system to Mn toxicity

**Fig. 3:**
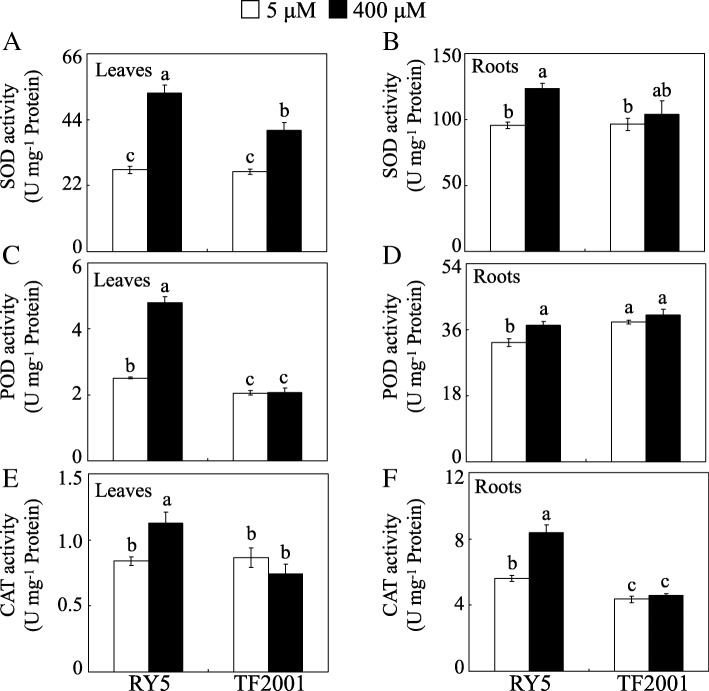
Analysis of antioxidant enzyme activities. SOD activity in leaves (**a**) and roots (**b**). POD activity in leaves (**c**) and roots (**d**). CAT activity in leaves (**e**) and roots (**f**). After 30 d of normal growth, stylo seedlings were treated with 5 or 400 μM MnSO_4_ for 10 d. Each bar indicates the mean of four biological replicates with standard error. The same letter represents no significant difference at the *P* = 0.05 level

Subsequently, the activities of three antioxidant enzymes, superoxide dismutase (SOD), peroxidase (POD) and catalase (CAT), were detected in both the leaves and roots of stylo under different Mn treatments. The results showed that SOD, POD and CAT activities in the leaves and roots of RY5 were significantly increased by excess Mn treatment, whereas only SOD activity was enhanced by Mn toxicity in the leaves of TF2001, compared to the respective controls (Fig. 3). The SOD, POD and CAT activities in the leaves of RY5 were increased by 94.0, 91.0 and 34.1% in the excess Mn treatment compared to those of their respective controls, and the activities of the three tested enzymes in the roots of RY5 were increased by more than 15.0% under excess Mn conditions compared to their respective controls (Fig. 3).

**Fig. 4:**
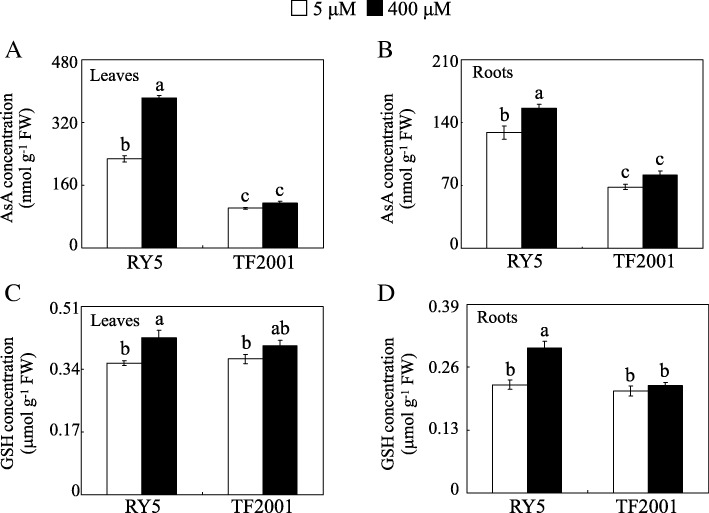
AsA and GSH concentrations in stylo in the two Mn treatments. AsA concentrations in leaves (**a**) and roots (**b**). GSH concentrations in leaves (**c**) and roots (**d**). Stylo seedlings were grown under normal conditions for 30 d and then treated with 5 or 400 μM MnSO_4_ for 10 d. Each bar indicates the mean of four independent replicates with standard error. The same letter represents no significant difference at the *P* = 0.05 level

Similarly, ascorbate (AsA) and glutathione (GSH) concentrations were increased by excess Mn treatment in RY5 but not in TF2001 (Fig. 4). Compared to their respective controls, the concentrations of AsA in the leaves and roots of RY5 increased by 68.4 and 21.0% under Mn toxicity, respectively, while the GSH concentrations in leaves and roots of RY5 increased by 19.2 and 33.8% at excess Mn levels, respectively (Fig. 4). These results suggest that the Mn-tolerant stylo genotype RY5 responds to Mn toxicity by enhancing the antioxidant system.

### Identification of differentially expressed proteins regulated by Mn toxicity in stylo

To dissect the molecular mechanisms underlying the response of stylo to Mn toxicity, a label-free proteomic approach was conducted to identify excess-Mn-responsive proteins in the leaves and roots of the Mn-tolerant stylo genotype RY5. A total of 3048 proteins were identified in both the leaves and roots of RY5 in the two Mn treatments, including 2623 and 2669 proteins detected from the leaves and roots, respectively, and 2244 proteins (73.6% of the total) overlapped between the leaves and roots (Additional file 2). Proteins with significant changes (*P* value<0.05) in abundance and abundance changes greater than 2-fold were defined as differentially expressed proteins (DEPs) regulated by excess Mn. In total, 356 proteins exhibited differential expression under the two Mn levels, including 206 proteins from the leaves and 150 proteins from the roots. These DEPs consisted of 71 upregulated, 62 downregulated, 127 strongly induced and 96 completely suppressed proteins (Additional file 2). Among these 356 DEPs, 195 proteins (54.8% of the total) were specific to the leaves, and 139 proteins (39.0% of the total) were unique to the roots, while 11 proteins (3.1% of the total) overlapped in the leaves and roots (Additional file 2). Detailed information on the fold changes and annotations of the DEPs from the leaves and roots is summarized in Additional file 3.

### Functional cataloging of Mn-responsive proteins

**Fig. 5:**
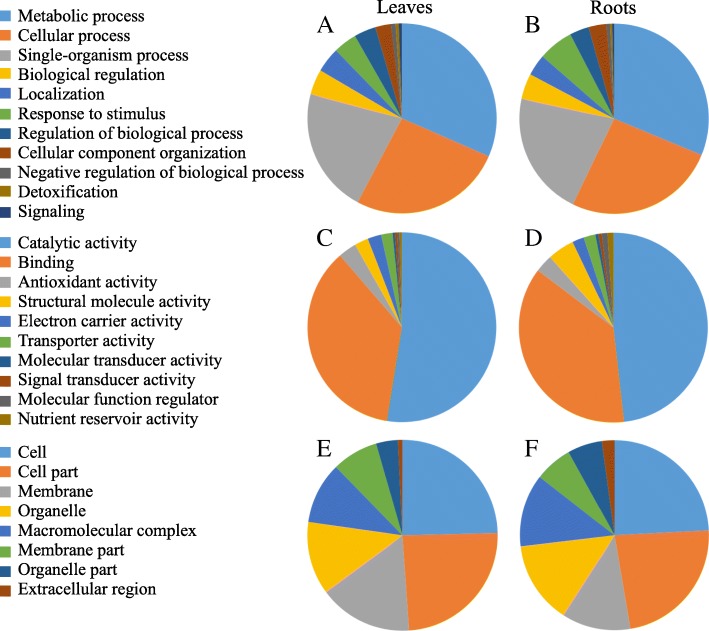
Gene ontology (GO) analysis of DEPs in stylo. DEPs in leaves (**a**, **c**, **e**) and roots (**b**, **d**, **f**) were classified into three groups: biological process (**a**, **b**), molecular function (**c**, **d**) and cellular component (**e**, **f**)

**Fig. 6:**
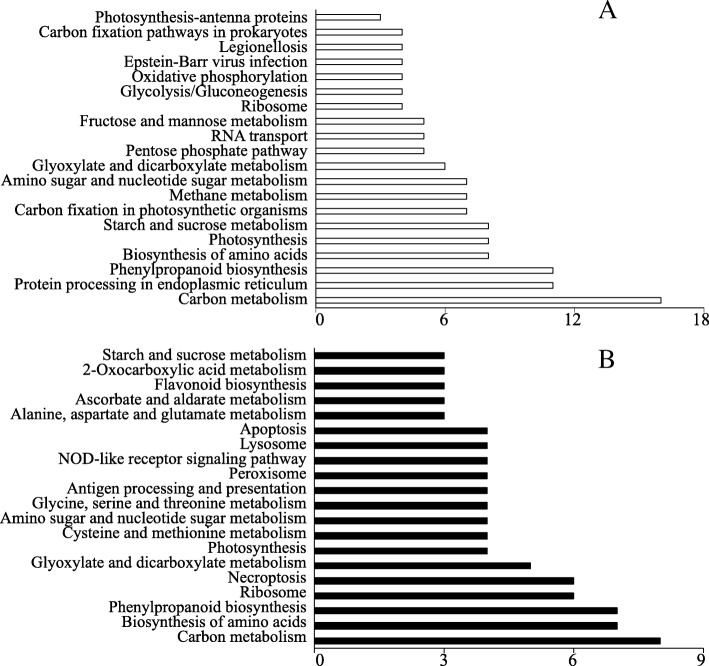
KEGG pathway enrichment analysis of the DEPs identified in the leaves (**a**) and roots (**b**) of stylo

To further investigate the proteomic changes of stylo in response to Mn toxicity, functional categories, including biological process (BP), molecular function (MF) and cellular component (CC), were determined according to the Gene Ontology (GO) database (Fig. 5). Because some of the DEPs were identified in multiple groups, out of the 356 DEPs, 261 proteins were classified into BP, 300 proteins were grouped into MF and 143 proteins belonged to CC (Fig. 5). The results showed that the functional classifications of the 356 DEPs were similar between leaves and roots. Among them, the dominant categories in BP included metabolic process, cellular process and single-organism process terms; the main MF categories were catalytic activity and binding terms; and the prominent categories in CC were cell, cell part and membrane terms (Fig. 5). Subsequently, functional classification of the DEPs was performed according to KEGG analysis. The results showed that the main KEGG pathways in leaves were carbon metabolism, protein processing in endoplasmic reticulum, phenylpropanoid biosynthesis, biosynthesis of amino acids, photosynthesis and starch and sucrose metabolism (Fig. 6a). In roots, the dominant KEGG pathways were carbon metabolism, biosynthesis of amino acids and phenylpropanoid biosynthesis (Fig. 6b).

### Expression profiles of DEPs involved in the response of stylo to Mn toxicity

**Fig. 7:**
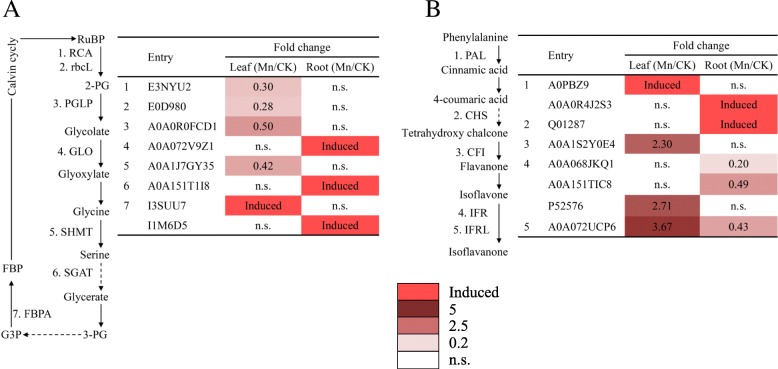
Expression profiles of DEPs involved in photorespiration cycle and phenylpropanoid pathway. (**a**) DEPs involved in the photorespiration cycle. (**b**) DEPs involved in the phenylpropanoid pathway. DEPs were mapped to the reference pathways in KEGG. Mn toxicity regulated fold changes of DEPs in the leaves and roots are shown

To detect the expression profiles of proteins involved in the response of stylo to Mn toxicity, candidate DEPs were selected and further separated into different functional groups, such as photosynthesis, carbon metabolism, TCA cycle, defense response, phenylpropanoid biosynthesis, protein metabolism, cell wall modulation and signaling (Table 2). The expression profiles of DEPs involved in photorespiration and the Calvin cycle in leaves and roots of stylo were then extracted and mapped to the reference pathways in KEGG. The results showed that rubisco activase (RCA), ribulose bisphosphate carboxylase large chain (rbcL), phosphoglycolate phosphatase (PGLP), and serine hydroxymethyltransferase (SHMT) were downregulated and fructose-bisphosphate aldolase (FBPA1) was induced in stylo leaves under excess Mn (Fig. 7a). In roots, peroxisomal (S)-2-hydroxy-acid oxidase (GLO), serine-glyoxylate aminotransferase (SGAT) and FBPA2 were strongly induced by Mn toxicity (Fig. 7a). Furthermore, the expression profiles of DEPs involved in the phenylpropanoid pathway were investigated. In leaves, phenylalanine ammonia-lyase (PAL1) was induced, and chalcone-flavonone isomerase (CFI), isoflavone reductase (IFR3) and isoflavone reductase-like protein (IFRL) were upregulated by Mn toxicity (Fig. 7b). In roots, PAL2 and chalcone synthase (CHS) were induced by Mn toxicity, whereas IFR1, IFR2 and IFRL were downregulated (Fig. 7b).

**Table 2:** Proteins and their fold changes in the leaves and roots of stylo under Mn toxicity

Accession number (^a^)	Protein name (^b^)	Protein abbreviation	Fold change	
			Leaf (Mn/CK)	Root (Mn/CK)
Photosynthesis				
I1LC20	Chlorophyll a-b binding protein	LHCa/b^1^	n.s.	Suppressed
A0A151RZX1	Chlorophyll a-b binding protein	LHCa/b^2^	0.46	n.s.
A0A1S3TRJ8	Chlorophyll a-b binding protein	LHCa/b^3^	0.33	n.s.
A0A1L5BWG5	Photosystem I P700 chlorophyll a apoprotein A1	PsaA	0.46	n.s.
V5JDR5	Photosystem I iron-sulfur center	PsaC	0.49	n.s.
D3J8F9	Photosystem II CP43 reaction center protein	PsbC^1^	0.29	n.s.
R9ZUR7	Photosystem II CP43 reaction center protein	PsbC^2^	Induced	n.s.
G7K9H5	Photosystem II oxygen-evolving enhancer protein	OEE	n.s.	Suppressed
A0A1S2XT02	Photosynthetic NDH subunit of subcomplex B 2	PNSB2	Suppressed	n.s.
A0A1S2YJB0	Electron transfer flavoprotein subunit alpha	ETFA	Suppressed	n.s.
Q9LKH8	NADPH-protochlorophyllide oxidoreductase	Por	n.s.	Suppressed
A0A0B2QNM0	ATP-dependent Clp protease ATP-binding subunit clpA like CD4B	CD4B	n.s.	Suppressed
V7BKQ7	Cytochrome c oxidase subunit 5C	COX5C	2.34	n.s.
E3NYU2	Rubisco activase 2	RCA	0.30	n.s.
E0D980	Ribulose bisphosphate carboxylase large chain	rbcL	0.28	n.s.
A0A0R0FCD1	Phosphoglycolate phosphatase	PGLP	0.50	n.s.
A0A072V9Z1	Peroxisomal (S)-2-hydroxy-acid oxidase GLO1	GLO	n.s.	Induced
A0A1J7GY35	Serine hydroxymethyltransferase	SHMT	0.42	n.s.
A0A151T1I8	Serine-glyoxylate aminotransferase	SGAT	n.s.	Induced
TCA cycle				
A0A0B2S2G8	Citrate synthase	CS	n.s.	2.04
A0A1S3VRH0	Succinate dehydrogenase	SDH	Induced	n.s.
G7JYQ8	Aconitate hydratase	AH^1^	Induced	n.s.
A0A1S2Z8R6	Aconitate hydratase	AH^2^	n.s.	0.47
Carbon fixation				
C9W980	Phosphoenolpyruvate carboxylase	PEPC^1^	2.58	n.s.
B0LXE5	Phosphoenolpyruvate carboxylase	PEPC^2^	2.75	n.s.
Q8H946	Phosphoenolpyruvate carboxylase	PEPC^3^	3.80	n.s.
Q8W4X0	Cytosolic malate dehydrogenase	MDH	Suppressed	n.s.
A0A0L9VHL1	Malic enzyme	ME	n.s.	Suppressed
Glycolysis				
I1M6D5	Fructose-bisphosphate aldolase	FBPA^1^	n.s.	Induced
I3SUU7	Fructose-bisphosphate aldolase	FBPA^2^	Induced	n.s.
V7C1L3	Glucose-6-phosphate isomerase	G6PI	Suppressed	n.s.
Amino acid metabolism				
O04998	Glutamine synthetase	GLN	2.49	n.s.
A0A1S3VJ34	Glutamate synthase	GLT	n.s.	0.33
A0A142F3D2	Glutamate dehydrogenase	GluDH	Induced	n.s.
A0A1S3TXT3	Ornithine carbamoyltransferase	OCT	n.s.	Induced
A0A1S3EC55	S-adenosylmethionine synthase	SAMS	2.37	n.s.
Other metabolism				
A0A0B2PML9	Sucrose synthase 2	SuSy	Suppressed	n.s.
A0A151TQQ3	Alde 1-epimerase	Aepi	4.18	n.s.
Q7X9T1	Alpha-amylase	AMY	Induced	n.s.
H6U596	Alcohol dehydrogenase	ADH	Induced	n.s.
B0FBK6	Acetolactate synthase	ALS	n.s.	Induced
A0A0B2NWN8	3-hydroxyisobutyryl-CoA hydrolase-like protein 5	3-HCHL	Suppressed	n.s.
A0A151R9N4	Formate dehydrogenase	FDH	Induced	n.s.
ATP synthase				
A0A1J7HQT2	ATP synthase subunit beta	ATP synthase^1^	n.s.	Induced
A0A1S2YAS0	ATP synthase subunit b	ATP synthase^2^	n.s.	Suppressed
A0A072TW18	F0F1-type ATP synthase	ATP synthase^3^	Induced	n.s.
A0A120IH31	ATPase subunit 8	ATPase	Induced	n.s.
Defense response				
A0A1J7HRM7	Peroxidase	POD^1^	2.95	n.s.
P22195	Cationic peroxidase 1	POD^2^	2.18	n.s.
A0A072UXA0	Peroxidase	POD^3^	Induced	n.s.
A0A0B2SVW0	Peroxidase	POD^4^	Induced	n.s.
A0A1J7G991	Peroxidase	POD^5^	Induced	n.s.
A0A151TL42	Peroxidase	POD^6^	Induced	n.s.
G7INV1	Peroxidase	POD^7^	Induced	n.s.
A0A151R770	Peroxidase	POD^8^	n.s.	0.45
K7KG78	Peroxidase	POD^9^	n.s.	2.70
I1LI46	Peroxidase	POD^10^	n.s.	Suppressed
A0A1S2Y825	Peroxidase	POD^11^	n.s.	Suppressed
A0A1S3T7B9	Peroxidase	POD^12^	n.s.	Suppressed
A0A1S2YK26	Probable glutathione S-transferase	GST	3.75	n.s.
Q5XXQ4	Pathogenesis-related protein 10	PR10^1^	n.s.	2.21
Q6VT83	Pathogenesis-related protein 10	PR10^2^	n.s.	0.10
K4LMW7	Pathogenesis-related protein 10b	PR10b	Induced	n.s.
Q7X9F6	Chitinase class Ib	CHI	Induced	n.s.
A0A151R8U8	Endochitinase	ECHI	Induced	n.s.
Q9S9H7	Beta-1,3-glucanase	GLU	n.s.	0.27
A2Q4Q4	Polyphenol oxidase	PPO	2.33	n.s.
G7K6C8	NAD(P)H:quinone oxidoreductase	NQOR	Induced	n.s.
A0A1S3VW46	Quinone oxidoreductase-like protein 2 homolog	QOR	Induced	n.s.
A0A1S3VNB5	Probable NAD(P)H dehydrogenase (Quinone) FQR1-like 2	FQR1L2	n.s.	Suppressed
A0A0K0K9R9	Universal stress protein	USP	Suppressed	Suppressed
G9C018	Cold dehydrin	CDD	Induced	n.s.
A0A151TJU2	Osmotin-like protein	Osmotin	Induced	3.46
A0A151T914	Light-regulated protein	LRP	0.37	n.s.
A0A151TUS7	Protein IN2–1 isogeny B	IN2–1	2.66	n.s.
Phenylpropanoid pathway				
A0PBZ9	Phenylalanine ammonia-lyase	PAL^1^	Induced	n.s.
A0A0R4J2S3	Phenylalanine ammonia-lyase	PAL^2^	n.s.	Induced
Q01287	Chalcone synthase 2	CHS	n.s.	Induced
A0A1S2Y0E4	Chalcone-flavonone isomerase family protein	CFI	2.30	n.s.
A0A068JKQ1	Isoflavone reductase	IFR^1^	n.s.	0.20
A0A151TIC8	Isoflavone reductase	IFR^2^	n.s.	0.49
P52576	Isoflavone reductase	IFR^3^	2.71	n.s.
A0A072UCP6	Isoflavone reductase-like protein	IFRL	3.67	0.43
A0A1S2YI24	2-hydroxyisoflavanone synthase-like	IFS	n.s.	Suppressed
Lignin pathway				
A0A1J7GGU7	Dirigent protein	DIR^1^	Induced	n.s.
A0A151SV45	Dirigent protein	DIR^2^	2.13	n.s.
A0A0L9VQD6	Dirigent protein	DIR^3^	n.s.	Induced
E3NYT4	Dirigent protein	DIR^4^	n.s.	2.36
Cytoskeleton				
A0A072VQC8	Tubulin alpha chain	α-tubulin	n.s.	Induced
P37392	Tubulin beta-1 chain	β-tubulin	n.s.	Induced
Cell wall modulation				
M1PNG4	Expansin	Exp	Suppressed	n.s.
G7IMV1	Alpha-L-arabinofuranosidase/beta-D-xylosidase	ASD/BXL	n.s.	3.73
A0A0B2PFS3	Fasciclin-like arabinogalactan protein 12	FLA	n.s.	0.50
A0A151SRS4	Xyloglucan endotransglucosylase/hydrolase	XTH	n.s.	2.01
A0A1S3UQ47	Plasmodesmata callose-binding protein 3	PDCB	Induced	2.17
Transcription				
A0A0B2Q3X5	Histone H1.2	H1.2	n.s.	2.31
C6TDQ3	Nuclear pore complex protein NUP35	NUP35	Induced	n.s.
RNA processing				
A0A151SA18	Ribonucleases P/MRP protein subunit POP1	POP1	n.s.	5.88
Protein synthesis				
A0A1S2X984	Elongation factor 1-gamma	EF1g	Induced	Induced
A0A072VFI9	Elongation factor 1-alpha	EF1a	Suppressed	n.s.
V7ATK1	Eukaryotic translation initiation factor 3 subunit B	eTIF3b	Induced	n.s.
A0A1J7G0E8	Eukaryotic translation initiation factor 3 subunit G	eTIF3g	2.19	n.s.
P17092	30S ribosomal protein S17	S17	2.23	n.s.
N0DM61	30S ribosomal protein S7	S7	n.s.	Suppressed
A0A1S2Z5B3	50S ribosomal protein L4	L4	n.s.	0.46
A0A191UJA2	50S ribosomal protein L14	L14	n.s.	Suppressed
Protein processing and transporting				
A0A1S2X9H9	Heat shock 70 kDa protein	HSP70	2.08	n.s.
G7IDZ4	Heat shock protein 81–2	HSP81–2	n.s.	2.29
I1L314	Heat shock protein 90–1	HSP90–1	n.s.	Induced
I1LD87	Protein disulfide isomerase-like 7	PDIL7	Induced	n.s.
A0A151T7Z7	UDP-glucose:glycoprotein glucosyltransferase 1	UGGT	2.56	n.s.
A0A1S2Z565	Dolichyl-diphosphooligosaccharide-protein glycosyltransferase subunit STT3B	STT3B	Induced	n.s.
Protein degradation				
A0A072TU80	Ubiquitin-conjugating enzyme	UBC	Induced	n.s.
A0A151TZ12	E3 ubiquitin-protein ligase UPL1	UPL1	Induced	n.s.
A0A1S2Z415	Leucine aminopeptidase 1-like	LAPL1	Suppressed	n.s.
Signaling				
A0A1S3VLK4	CBS domain-containing protein CBSX1	CBSX1	Induced	n.s.
A0A072UP16	Osmosensor histidine kinase	OHK	n.s.	Induced
A0A151TSS1	14–3-3 like protein B	14–3-3	2.21	n.s.
A0A151SLL9	Putative inactive receptor kinase	IRK	Induced	n.s.
A0A0L9U108	Serine/threonine-protein phosphatase	PP	Suppressed	n.s.
A0A072UKS2	PfkB family carbohydrate kinase	PfkB	Suppressed	n.s.
G7JHI0	General regulatory factor 2	GRF2	Suppressed	n.s.
I3S111	RAB GTPase-like protein A1D	A1D	n.s.	Suppressed
G7L1E7	C2 calcium/lipid-binding and GRAM domain protein	CalBP	n.s.	Suppressed

Note: ^a^Database accession number from UniProt; ^b^The name and functional categories of the proteins identified by LC-MS/MS. Stylo seedlings were grown under normal conditions for 30 d and then treated with 5 (CK) or 400 μM (Mn) MnSO_4_ for 10 d. Differentially expressed proteins in the leaves and roots of RY5 regulated by Mn toxicity were identified using a label-free quantitative proteomics approach. n.s. represents no significant difference

### qRT-PCR analysis of genes encoding the candidate DEPs

**Fig. 8:**
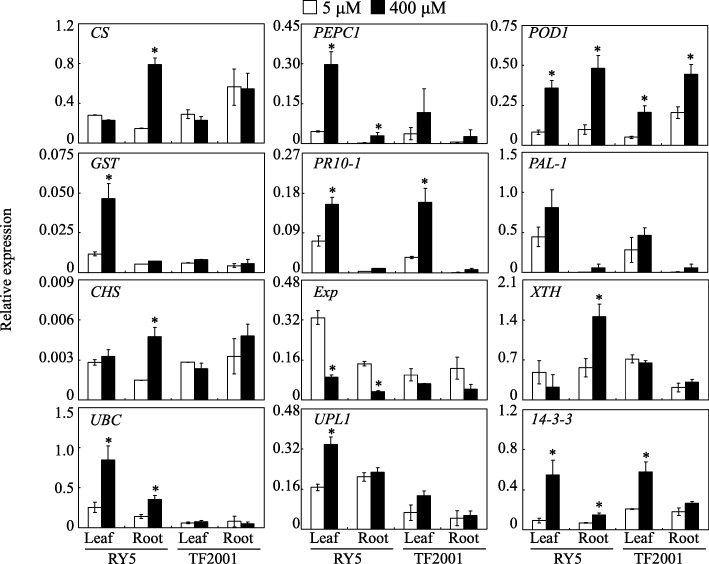
Transcription levels of genes encoding excess Mn regulated proteins. qRT-PCR was performed to detect gene expression in stylo leaves and roots treated with 5 or 400 μM MnSO_4_. Each bar indicates the mean of three independent replicates with standard error. Asterisks indicate significant differences between 5 μM and 400 μM Mn treatments at the *P* = 0.05 level

Subsequently, the expression levels of 12 selected genes encoding the candidate DEPs were further detected using qRT-PCR analysis. As shown in Fig. 8, the transcriptional levels of 10 out of 12 genes in RY5 leaves or roots were consistent with their protein accumulation patterns after Mn exposure. Among them, the expressions of *citrate synthase* (*CS*), *phosphoenolpyruvate carboxylase* (*PEPC1*), *peroxidase* (*POD1*), *probable glutathione S-transferase* (*GST*), *chalcone synthase* (*CHS*), *xyloglucan endotransglucosylase*/*hydrolase* (*XTH*), *ubiquitin*-*conjugating enzyme* (*UBC*), *E3 ubiquitin*-*protein ligase* (*UPL1*) and *14–3-3* were increased in RY5 leaves or roots with excess Mn treatments, while the *expansin* (*Exp*) gene was downregulated in leaves and roots of RY5 under Mn toxicity compared to those of the controls (Fig. 8). However, the transcriptional levels of *pathogenesis*-*related protein* 10 (*PR10–1*) and *phenylalanine ammonia*-*lyase* (*PAL1*) differed from the accumulation patterns of their respective encoding proteins in the RY5 response to Mn treatments (Fig. 8). Furthermore, except for *POD1*, *PR10–1* and *14–3-3*, the transcripts of the other tested genes were not regulated by excess Mn in leaves or roots of TF2001 (Fig. 8).

## Discussion

### RY5 has superior Mn tolerance

Mn toxicity disrupts various biological processes in plants, such as inhibiting chlorophyll biosynthesis and photosynthesis, which decreases plant yield [11, 13]. Plants originating from tropical and subtropical areas are assumed to have great potential to tolerate metal toxicity, such as Al and Mn toxicity [25, 29, 30]. In accordance with this hypothesis, among 12 different legume plants, stylo, a tropical legume, exhibits high Mn tolerance [27]. However, the physiological and molecular mechanisms underlying the response of stylo to Mn toxicity are poorly understood. In this study, variations in Mn tolerance were observed among nine stylo genotypes (Additional file 1). RY5, a Mn-tolerant genotype, and TF2001, a Mn-sensitive stylo genotype, were further selected to dissect Mn toxicity/tolerance mechanisms. The results showed that when grown at 400 μM Mn, the stylo genotype RY5 exhibited higher Mn tolerance than the genotype TF2001, as reflected by the smaller reduction in leaf chlorophyll concentration, chlorophyll fluorescence parameters, photosynthetic indexes and the maintenance of RY5 plant dry weight (Table 1 and Fig. 1). Similar results have been found in perennial ryegrass; the plant dry weight, chlorophyll concentration and photosynthesis declined to a greater extent in the Mn-sensitive cultivar Nui than in the Mn-tolerant cultivar Kingston under Mn toxicity [13]. Furthermore, although the Mn concentrations in the shoots and roots were significantly increased in both stylo genotypes subjected to Mn toxicity, the Mn levels in the shoots and roots were lower in RY5 than in TF2001 under excess Mn treatment (Fig. 1). This result suggests that the regulation of Mn uptake and transport might be a strategy for adapting to Mn toxicity. Similarly, regulation of Mn homeostasis has also been reported in the rice response to Mn toxicity [31].

**Fig. 9:**
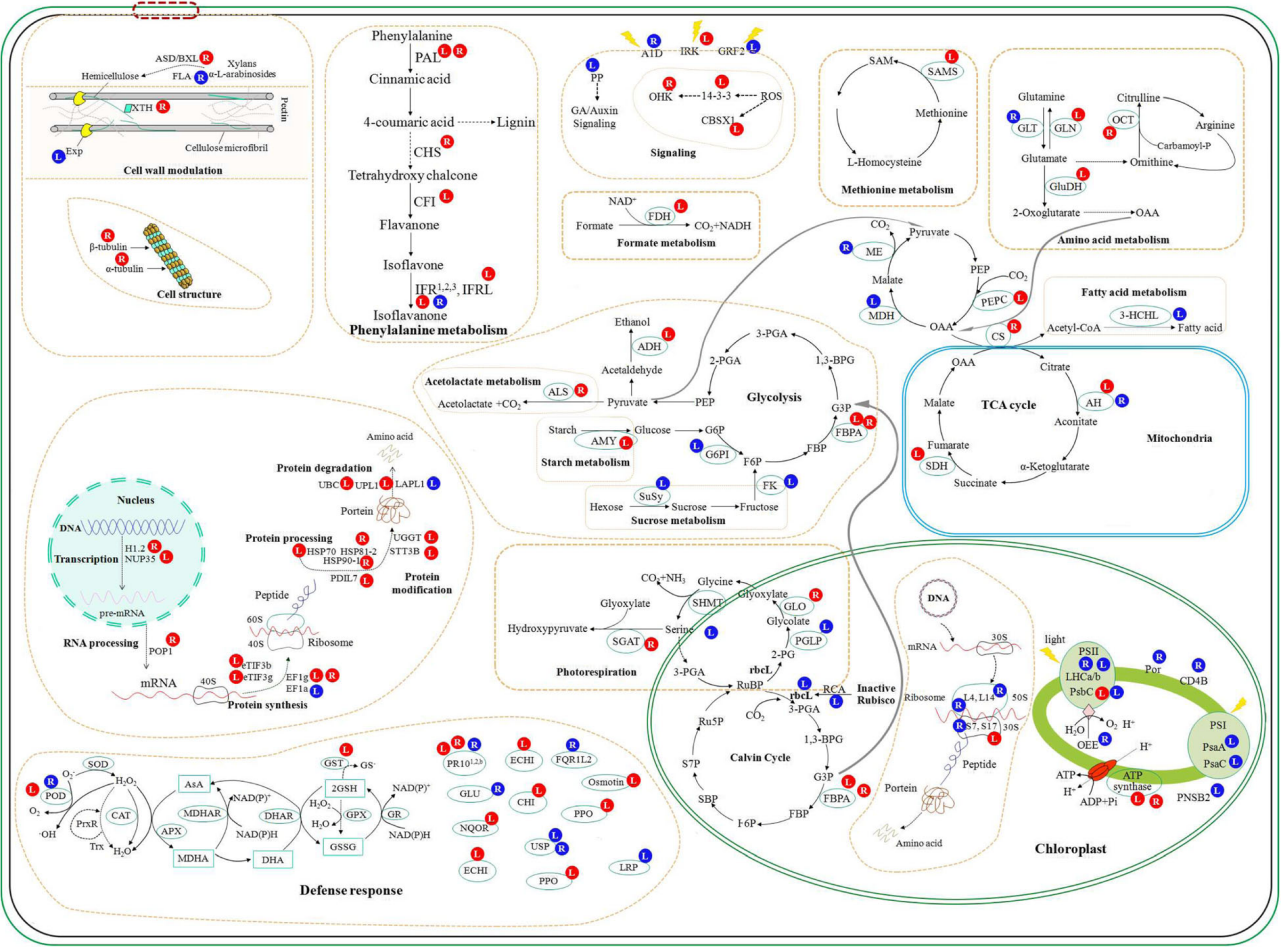
Schematic presentation of molecular processes in the response of stylo to Mn toxicity. The identified proteins were integrated into specific pathways. Protein expression patterns in leaves (L) and roots (R) are shown in red (increased) or blue (decreased), respectively

Although excess-Mn-regulated DEPs have been previously reported in different plant species, such as cowpea [17], soybean [32], *Citrus* [33], rice and barley [34], this study identified many additional Mn-regulated DEPs using a label-free proteomic approach. Furthermore, the candidate DEPs in the response of stylo to Mn toxicity were integrated into specific pathways, and a schematic was constructed to display the molecular processes in the leaves and roots of stylo in response to Mn toxicity (Fig. 9 and Table 2).

### Photosynthesis modulation under Mn toxicity

Mn toxicity-caused inhibition of plant growth is closely related to the reduction of photosynthesis [12, 35, 36]. Under excess Mn condition, the photosynthesis of stylo was significantly inhibited, which was reflected by the decline of chlorophyll content, fluorescence parameters (e.g., *F*v/*F*m, ΦPSII, 1-qL and ETR) and photosynthetic indexes (e.g., Pn, Ci and Gs), especially in the Mn-sensitive stylo genotype TF2001 (Table 1). Similar phenomenon were also observed in rice [35], *Citrus* [12], polish wheat [36] and cucumber (*Cucumis sativus*) [37] exposed to Mn toxicity.

The stability and activity of PSII were probably affected in stylo by Mn toxicity, as reflected by the decline of *F*v/*F*m, ΦPSII and 1-qL and ETR (Table 1), and the decreased abundances of PSII complex proteins, such as PSII CP43 reaction center protein (PsbC), PSII oxygen-evolving enhancer protein (OEE) and chlorophyll a-b binding protein (LHCa/b) (Table 2). As LHCa/b and PsbC are components of the PSII complex and help catalyze the light-induced photochemical processes of PSII [38], the decrease in the abundance of LHCa/b and PsbC suggests that the PSII photochemistry activity is modulated in the response of stylo to Mn toxicity. Furthermore, the decreased abundances of electron transfer flavoprotein subunit (ETFA) and PSI complex proteins, such as PSI P700 chlorophyll a apoprotein A1 (PsaA) and PSI iron-sulfur center (PsaC), would lead to inhibit electron transport process in stylo (Fig. 9 and Table 1). Similarly, decreases in PsaA protein levels have also been observed in Arabidopsis treated with excess Mn [9]. Besides, the regulation of LHCa/b levels is regarded as an adaptive strategy to balance energy distribution between PSII and PSI for redox homeostasis modulation in chloroplasts [39]. Thus, the PSI and PSII complexes are likely the major targets of Mn toxicity in stylo.

In this study, Gs, Ci and Pn significantly declined in stylo under excess Mn condition (Table 1). The declined stomatal conductance probably led to the decreased leaf intercellular CO_2_ levels and consequently the decreased photosynthesis in stylo. This was supported by subsequent proteomics analysis that decreases in the abundances of photorespiration- and Calvin cycle-related proteins were observed in the leaves of RY5 treated with excess Mn, including rubisco activase (RCA), ribulose bisphosphate carboxylase large chain (rbcL), phosphoglycolate phosphatase (PGLP), and serine hydroxymethyltransferase (SHMT) (Fig. 7 and Table 2). RCA and rbcL catalyze the most crucial step of carbon fixation within the photorespiration and Calvin cycle pathways. The reduction of photosynthesis in white birch (*Betula platyphylla*) under Mn toxicity has been attributed to the modification of rubisco and the inhibition of ribulose-1,5-bisphosphate regeneration through the Calvin cycle [40]. Furthermore, PGLP dephosphorylates the 2-phosphoglycolate produced by rubisco to generate glycolate, which can be further catalyzed by peroxisomal (S)-2-hydroxy-acid oxidase (GLO) to produce glyoxylate, which facilitates glycine generation [41, 42]. SHMT catalyzes the interconversion of glycine and serine and is involved in controlling cell damage caused by salt and hypersensitive stress [43]. Interestingly, the abundance of fructose-bisphosphate aldolase (FBPA), which is involved in glycolysis and the Calvin cycle, was enhanced in leaves and roots of stylo under Mn toxicity (Fig. 7 and Table 2). The changes in abundance of the above proteins probably modulate the reduction of photosynthesis in the response of stylo to Mn toxicity.

### Defense response of RY5 to Mn toxicity

In this study, accumulation of H_2_O_2_ and MDA was increased in the leaves and roots of stylo treated with excess Mn, especially in TF2001 (Fig. 2). Such accumulation may result in cellular damage through oxidative stress. The elimination of excess ROS stress via regulation of the antioxidant system is employed by plants to manage Mn toxicity [11, 17]. Excess Mn increased the activities of SOD, POD and CAT (Fig. 3) and the concentrations of AsA and GSH in RY5 (Fig. 4), suggesting that antioxidant enzymes and the AsA-GSH cycle are most likely involved in ROS scavenging in stylo. Consistent with the increased POD activity under excess Mn, the abundances of seven PODs and *POD1* gene transcripts were enhanced in stylo leaves under Mn toxicity, although other POD homologues exhibited different regulation in roots (Fig. 8 and Table 2). An increased accumulation of PODs has been found to modulate Mn oxidation and compartmentalization in cowpea [17].

Other defense response proteins, such as glutathione S-transferase (GST), pathogenesis-related proteins (PRs), chitinase (CHI) and polyphenol oxidase (PPO), were identified in this study (Table 2). Homologues of these proteins have been demonstrated to respond to abiotic stresses. For example, increased protein abundance of GST has been reported in roots of Arabidopsis under cadmium (Cd) treatment [44]. Overexpression of *GST* in Arabidopsis decreases ROS accumulation under salinity, improving salt stress tolerance [45]. Furthermore, CHI acts as one of the second-line defense components involved in plant metal stress tolerance by affecting the metal binding and immobilization capability of the cell wall [46]. In addition, as a stress-related protein, PPO catalyzes the oxidation of polyphenols into quinones, and the expression of PPO is enhanced under Cd and mercury (Hg) treatments [45]. Thus, enhancement of ROS scavenging and defense response may be important strategies for RY5 to counteract injuries arising from Mn-provoked oxidative damage.

### Metabolism adjustment under Mn toxicity

Metabolic processes have been found to respond to Mn toxicity in many plants [8]. In this study, a set of excess Mn-regulated DEPs were associated with specific pathways (Table 2), such as the TCA cycle, carbon fixation, amino acid and other metabolism, suggesting that complex changes arise in stylo during adaptation to Mn toxicity. Several proteins involved in the TCA cycle, including citrate synthase (CS), aconitate hydratase (AH) and succinate dehydrogenase (SDH), were differentially regulated by Mn toxicity in the leaves and roots of stylo (Fig. 9). CS catalyzes the combination of oxaloacetate (OAA) and acetyl CoA to produce citrate, which can isomerize into isocitrate via cis-aconitate catalyzed by AH [47]. Furthermore, several proteins involved in carbon fixation, including phosphoenolpyruvate carboxylase (PEPC), malate dehydrogenase (MDH) and malic enzyme (ME), were regulated by Mn toxicity in stylo leaves and roots in this study (Fig. 9). PEPC has been demonstrated to irreversibly catalyze the conversion of phosphoenolpyruvate (PEP) to OAA, which is catalyzed by MDH to produce malate; malate is then catalyzed by ME to generate pyruvate [48]. Although important roles of organic acids (e.g., citrate and malate) in Mn detoxification are suggested in some plants, including cowpea, ryegrass, white clover and *Phytolacca americana* [23, 49, 50], only one MDH has been found to regulate malate synthesis and exudation, conferring high Mn tolerance in stylo [25]. Thus, the potential roles of the candidate DEPs described above in the management of Mn toxicity in stylo through the regulation of organic acid metabolism warrant further study.

Additionally, amino acid metabolism-related proteins, such as glutamine synthetase (GLN) and glutamate synthase (GLT) and glutamate dehydrogenase (GluDH), were identified in this study (Table 2). Furthermore, S-adenosylmethionine synthetase (SAMS), which catalyzes the formation of SAM, a compound involved in the biosynthesis of sulfur amino acids, polyamines and ethylene [51], was found to be upregulated by Mn toxicity in the leaves of stylo (Fig. 9). The expression of SAMS has also been found to be modulated by arsenic treatments [52]. Therefore, our results suggest that adjustment of amino acid metabolism might be important in the response of stylo to Mn toxicity.

### DEPs involved in the phenylpropanoid pathway

Although several secondary metabolites have been reported to be regulated by excess Mn in plants, such as phenolics and callose in cowpea [17], lignin and flavonoids in rice [35] and phenylalanin in *Populus cathayana* [53], no phenylpropanoid pathway-related proteins have previously been identified using proteomic techniques. In the present study, key proteins involved in the phenylpropanoid pathway, including phenylalanine ammonia-lyase (PAL), chalcone synthase (CHS), chalcone-flavonone isomerase family protein (CFI), and isoflavone reductase (IFR), were found to be upregulated in the leaves or roots of stylo under Mn toxicity, whereas the other homologues of IFR were downregulated in the roots (Fig. 7 and Table 2). PAL is the primary enzyme in the phenylpropanoid pathway, of which the products, such as flavonoids and lignins, are key compounds in abiotic stress tolerance [54]. CHS is involved in the synthesis of tetrahydroxy chalcone, whereas CFI catalyzes the intramolecular cyclization of bicyclic chalcones into tricyclic (S)-flavanones, and IFRs then catalyze the reduction from achiral isoflavones to chiral isoflavanones [55, 56]. The expression levels of *PAL*, *CHS*, and *IFR* are enhanced by Cd and lead treatment as well as by salinity stress [57–59]. Similarly, in this study, the *CHS* transcript was also increased by excess Mn in RY5 roots (Fig. 8). The results of the present study suggest that the phenylpropanoid pathway is enhanced in stylo adaptation to excess Mn toxicity. This novel hypothesis merits further study.

### DEPs involved in cytoskeleton and cell wall modulation

In this study, cytoskeleton-related proteins, such as tubulin alpha chain (α-tubulin) and tubulin beta-1 chain (β-tubulin), and cell-wall-associated proteins, such as expansin (Exp), were regulated by Mn toxicity in stylo (Table 2). Tubulin is the major constituent of microtubules, which are the basic components of the cytoskeleton and participate in cell division and elongation. Suppression of *α-tubulin* results in decreased panicle elongation during drought stress in rice [60]. Expansin plays important roles in the regulation of cell wall extension and expansion [61]. Inhibition of plant growth is observed in tobacco overexpressing *TaEXPB23* from wheat and in tomato overexpressing *CsExp1* from *Cucumis sativus* [61, 62]. Interestingly, another cell-wall-associated protein, xyloglucan endotransglucosylase/hydrolase (XTH), was found, for the first time, to be upregulated by Mn toxicity in stylo roots (Table 2). Furthermore, *XTH* expression level was increased by Mn toxicity in RY5 roots (Fig. 8). XTH catalyzes xyloglucan endohydrolysis (XEH) and/or endotransglycosylation (XET), thereby controlling cell wall extension and hemicellulose accumulation [63]. It has been reported that disruption of *XTH31* in Arabidopsis results in higher Al resistance than in wild type, as reflected by reduced inhibition of root growth under Al toxicity [64]. Therefore, the regulation of proteins associated with the cytoskeleton and cell wall structure may be important for root growth in stylo exposed to Mn toxicity.

### Transcriptional regulation and protein turnover in stylo

In this study, two transcription-related proteins, one nuclear pore complex protein NUP35 (NUP35) in leaves and one histone H1.2 (H1.2) in roots, were upregulated in stylo under Mn toxicity (Fig. 9 and Table 2). The nuclear pore complex facilitates the exchange of macromolecules between the cytoplasm and nucleoplasm [65]. Histones play a central role in transcriptional regulation, DNA repair and chromosomal stability and are involved in stress tolerance in plants [66]. In addition, the abundance of one RNA processing-related protein, ribonuclease P/MRP protein subunit POP1 (POP1), which is involved in the generation of mature tRNA molecules by cleaving their 5’ ends [67], was increased in the roots of stylo under Mn toxicity (Fig. 9 and Table 2). The transcriptional regulation and RNA processing might be activated in the leaves and roots of stylo under Mn toxicity.

Several proteins involved in protein synthesis were identified in the leaves or roots of stylo (Fig. 9 and Table 2). For example, two elongation factors (e.g., EF1a and EF1g) involved in the promotion of GTP-dependent binding of aminoacyl-tRNA to ribosomes [68], and two eukaryotic translation initiation factors (e.g., eTIF3b and eTIF3g) that stimulate the binding of mRNA and methionyl-tRNAi to the 40S ribosome [69] were regulated by Mn toxicity in stylo leaves and roots (Fig. 9 and Table 2). Furthermore, the accumulation of two proteins involved in protein degradation, ubiquitin-conjugating enzyme (UBC) and E3 ubiquitin-protein ligase (UPL1), was induced in stylo leaves by Mn toxicity (Table 2). Interestingly, the expression levels of *UBC* and *UPL1* were also found to be increased under excess Mn conditions in RY5 leaves (Fig. 8), suggesting that ubiquitin-dependent protein degradation is activated in the RY5 response to Mn toxicity. Although the exact functions and mechanisms of these DEPs involved in protein synthesis and degradation remain largely unknown, our results suggest that the regulation of protein turnover might be an important process in the response of stylo to Mn toxicity. Future investigation of the functions of these proteins will help reveal the mechanisms of high Mn tolerance in stylo.

### DEPs involved in signal sensing and transduction

In this study, several proteins associated with specific signaling pathways, including CBS domain-containing protein CBSX1 (CBSX1), osmosensor histidine kinase (OHK), serine/threonine protein phosphatase (PP), and 14–3-3 like protein (14–3-3), were found to be regulated by Mn toxicity in stylo (Fig. 9 and Table 2). For example, 14–3-3 proteins are involved in various metabolic processes and signaling pathways through interactions with numerous proteins in plant cells [70]. The OHK protein functions as an osmosensor and transmits stress signals to a downstream mitogen-activated protein kinase (MAPK) cascade [71]. Overexpression of *ATHK1*, a histidine kinase, results in increased water stress tolerance in Arabidopsis by regulating ABA signaling [72]. In addition, serine/threonine PP possesses phosphatase activity toward para-nitrophenyl phosphate (pNPP) in vitro and acts as a positive regulator in the gibberellin (GA) and auxin signaling pathways, which regulate plant growth and development [73]. Therefore, a sophisticated signaling network may be involved in the response of stylo to Mn toxicity, and further investigation of these proteins is required.

## Conclusion

In conclusion, the present study demonstrated that the stylo genotype RY5 had Mn tolerance superior to that of TF2001. The capability of RY5 to tolerate Mn toxicity was related to its reduced Mn absorption and stimulation of antioxidant protection, which in turn alleviated oxidative stress. The physiological and proteomic results suggest that a coordinately regulated accumulation of proteins associated with ROS scavenging, defense response, the phenylpropanoid pathway and protein metabolism helps stylo tolerate Mn toxicity. This study not only lays a foundation for further study of Mn tolerance mechanisms in tropical legumes but also provides candidate gene resources for breeding crop varieties tolerant to Mn toxicity through genetic improvement approaches.

## Methods

### Plant growth and treatments

In this study, nine stylo (*Stylosanthes guianensis*) genotypes, selected from the colony of its parent ‘CIAT184’ [74], were used to analyse their variability of Mn tolerance. The stylo seeds were provided by the Tropical Pasture Research Center, Institute of Tropical Crop Genetic Resources, Chinese Academy of Tropical Agricultural Sciences, Hainan Province, China. After seed germination for 3 d, stylo seedlings were transferred to a hydroponic box with 12 L of Hoagland’s nutrient solution [25]. The nutrient solution pH was adjusted to 5.8 using H_2_SO_4_ or KOH. Experiments were conducted in a greenhouse at the Institute of Tropical Crop Genetic Resources, Chinese Academy of Tropical Agricultural Sciences, Hainan Province, China (19°30′N, 109°30′E). After 30 d of growth, the uniform seedlings were transferred to fresh Hoagland’s nutrient solution supplied with 5 or 400 μM MnSO_4_ (pH 5.0) as previously described [25]. Stylo plants treated with 5 μM MnSO_4_ were used as the control. After 10 d of Mn treatments, both the shoots and roots were separately measured.

Two stylo genotypes contrasting in Mn tolerance, ‘RY5’ and ‘TF2001’, were selected for further analysis. After 30 d of growth, the uniform seedlings were subjected to 5 or 400 μM MnSO_4_ (pH 5.0) treatments. After 10 d of Mn treatments, both the shoots and roots were separately harvested for further analysis. Each treatment included four biological replicates. An individual hydroponic box containing three seedlings of each stylo genotype was considered one biological replicate.

### Determination of chlorophyll concentration and chlorophyll fluorescence parameters

Chlorophyll concentration in the leaves of stylo was determined according to the SPAD value measured by SPAD502 (Top Instruments Inc., China). Four chlorophyll fluorescence parameters, including *F*v/*F*m,ΦPSII, 1-qL and ETR, were measured by a pulse-modulated fluorometer Model FMS-2 (Hansatech Instruments Ltd., UK) according to the manufacturer’s instructions. The leaves were dark-adapted for 20 min before *F*v/*F*m analysis. *F*v/*F*m was calculated as *F*v/*F*m = (*F*m–*F*o)/*F*m, where *F*o and *F*m are the initial fluorescence yield and the maximum fluorescence yield, respectively. The leaves were then illuminated with actinic light. ΦPSII was calculated as ΦPSII = (*F*m′–*F*s)/*F*m′, where *F*m′ and *F*s are the maximal and steady-state fluorescence yields in a light-adapted state, respectively. 1-qL was calculated as 1-qL = 1–(*F*m′–*F*s)/(*F*m′–*F*o’)*·F*o’/*F*s, where *F*o’ is the minimal fluorescence yields in a light-adapted state [75]. ETR was calculated as ETR = ΦPSII·PPFD·0.84·0.5, where PPFD is an incident photosynthetic photon flux density.

### Determination of photosynthetic indexes

The Pn, Ci and Gs values were measured at 9:00 a.m. using a portable photosynthesis system LI-6400XT (LI-COR, USA) according to the manufacturer’s instructions. All the measurements were conducted at a constant flow rate of 400 μmol s^− 1^ and at saturation irradiance with PPFD of 1000 μmol m^− 2^ s^− 1^. Leaf temperature was controlled at 30 °C, and the CO_2_ concentration was set at 385 μmol CO_2_ mol^− 1^.

### Determination of Mn concentrations

Shoot and root samples were oven dried at 105 °C for 30 min and further dried at 75 °C. After determination of the shoot and root dry weight, the samples were ground into powder. Then, approximately 0.07 g samples were burned to ash in a muffle furnace at 600 °C for 10 h. After that, the sample ash was thoroughly dissolved in 7 mL of 100 mM HCl. Mn concentrations were determined using atomic absorption spectroscopy. A standard curve was used to quantify the Mn concentration in each sample as previously described [25].

### Analysis of H_2_O_2_ and MDA

H_2_O_2_ and MDA were measured using commercial chemical assay kits (Comin Ltd., China) as previously described [76]. Leaf and root samples were ground into powder in liquid nitrogen. H_2_O_2_ determination was carried out according to the assay kit, which was based on the titanium sulfate reaction. Briefly, approximately 0.1 g samples were extracted in 1 mL of acetone at 4 °C. The homogenates were centrifuged at 12,000×*g* for 10 min at 4 °C, and the supernatants were then collected. Subsequently, 250 μL supernatant was mixed with 25 μL buffer II and 50 μL buffer III from the assay kit. The mixture was centrifuged at 4000×*g* for 10 min at 25 °C, and the resulting pellets were then collected and dissolved in buffer IV from the assay kit. The H_2_O_2_ concentration was spectrophotometrically detected at 415 nm with a spectrophotometer (UV-2010, Hitachi, Japan), and calculated by comparison with a standard curve.

For MDA determination, approximately 0.1 g samples were ground in 1 mL of extraction buffer I from the assay kit at 4 °C, which was based on the thiobarbituric acid (TBA) reaction. The homogenates were centrifuged at 12,000×*g* for 10 min at 4 °C, and the supernatants were then collected. 0.1 mL supernatant was homogenized into 0.3 mL of a reaction buffer II from the assay kit. The mixture was heated at 95 °C for 30 min, immediately cooled in an ice bath, and centrifuged at 12,000×*g* for 10 min at 25 °C. The MDA concentration was determined at 532 nm and 600 nm, and calculated according to the instructions of manufacturers in the assay kit.

### Determination of enzyme activities and AsA and GSH concentrations

The activities of SOD, POD and CAT were measured using commercial chemical assay kits (Comin Ltd., China). Approximately 0.1 g of leaf and root samples were ground in 1 mL of extraction buffer from their respective assay kits at 4 °C. After centrifugation at 12,000×*g* for 10 min at 4 °C, the supernatants were collected and used for determination of the enzyme activities [76]. Briefly, SOD activity was detected based on the nitroblue tetrazolium chloride (NBT) reaction. 36 μL supernatant was mixed with 90 μL buffer I, 200 μL buffer II, 4 μL buffer III and 70 μL buffer IV from the assay kit. The reaction mixture was exposed to light for 30 min, and then the absorbance was measured at 560 nm with a spectrophotometer (UV-2010, Hitachi, Japan). The SOD activity was determined according to the instructions of manufacturers. POD activity was determined based on the guaiacol method. The reaction buffer contained 20 μL supernatant, 240 μL buffer I, 60 μL buffer II, 60 μL buffer III and 120 μL dH_2_O from the assay kit. POD activity was determined at 470 nm within 1 min, and defined as an absorbance change of 0.01 units per min. For CAT activity determination, the reaction buffer contained 20 μL supernatant and 380 μL buffer I from the assay kit. The CAT activity was determined at 240 nm within 1 min, and defined as the amount of protein required to oxidize 1 nmol H_2_O_2_ per min. The protein content in each sample was measured according to Bradford [77]. The specific enzyme activities were expressed as U mg^− 1^ protein.

For AsA and GSH determination, approximately 0.1 g of leaf and root samples were ground in 1 mL of extraction buffer from their respective assay kits (Comin Ltd., China) at 4 °C. The homogenates were centrifuged at 12,000×*g* for 15 min at 4 °C, and the supernatants were then collected for AsA and GSH analysis [76]. For AsA determination, the reaction buffer contained 40 μL supernatant, 320 μL buffer I and 40 μL buffer II from the assay kit. The AsA concentration was determined at 265 nm within 1 min, and calculated by comparison with a standard curve. For GSH analysis, the reaction buffer contained 40 μL supernatant, 280 μL buffer I and 80 μL buffer II from the assay kit. After incubation at 30 °C for 5 min, the GSH concentration was determined at 412 nm. A standard curve was used to quantify the GSH concentration in each sample. For blank controls, dH_2_O was used instead of supernatant in the reaction buffer. All of the experiments included three biological replicates.

### Label-free quantitative proteomics

The label-free proteomic approach was conducted at Shanghai Applied Protein Technology Company (APT Ltd., China). For protein extraction, leaf and root proteins were extracted using a previously described method [25]. Briefly, approximately 3 g samples were homogenized in modified Tris-HCl (pH 8.8) extraction buffer. After the homogenates were centrifuged, the supernatants were incubated with methanol at − 20 °C. After centrifugation, the resulting pellets were washed with acetone and ethanol. Finally, the resulting proteins were dissolved in SDT buffer (4% SDS, 100 mM DTT, 150 mM Tris-HCl, pH 8.0). The protein content of each sample was determined using the BCA Protein Assay Kit (Bio-Rad, USA).

For filter-aided sample preparation, 200 μg of protein extract for each sample was mixed with UA buffer (8 M urea, 150 mM Tris-HCl, pH 8.0). The detergent, DTT and other components in the mixture were removed by repeated ultrafiltration (Microcon units, 10 kD). Then, the proteins were alkylated with 100 μL of 100 mM iodoacetamide in UA buffer at room temperature for 30 min in the dark. The filters were washed three times with 100 μL of UA buffer and then twice with 100 μL of 25 mM NH_4_HCO_3_ buffer. The proteins were then digested with 4 μg trypsin (Promega, WI) in 40 μL of 25 mM NH_4_HCO_3_ buffer overnight at 37 °C, and the resulting peptides were recovered by centrifugation. The peptides of each sample were desalted on C18 Empore™ SPE Cartridges (Sigma, Germany) and concentrated by vacuum centrifugation, which was followed by reconstitution in 40 μL of 0.1% (v/v) formic acid.

For mass spectrometry, the peptide mixture was loaded onto a 2 cm × 100 μm reversed-phase C18 trap column (Thermo Fisher Scientific, Schwerte, Germany) and separated on a 10 cm × 75 μm C18 analytical column (Thermo Fisher Scientific) in mobile phases A (0.1% formic acid) and B (84% acetonitrile and 0.1% formic acid) at a flow rate of 300 nL/min. The gradient was increased to 55% of buffer B in 110 min and to 100% of buffer B in 5 min, with a hold at 100% of buffer B for 5 min. LC-MS/MS analysis was performed on a Q Exactive mass spectrometer (Thermo Fisher Scientific, Schwerte, Germany) coupled to an Easy nLC system (Thermo Fisher Scientific) for 120 min. MS data were acquired using a data-dependent top 10 method, dynamically choosing the most abundant precursor ions from the survey scan (300–1800 m/z). The resolution was set to 70,000 for the MS scans and 17,500 for the data-dependent MS/MS scans. The raw data have been deposited to the ProteomeXchange Consortium (http://proteomecentral.proteomexchange.org) via the PRIDE [78] partner repository with the dataset identifier PXD012379.

For protein identification and quantification, the MS data were analyzed using MaxQuant software version 1.5.3.17 (Max Planck Institute of Biochemistry in Martinsried, Germany). Database searches were conducted against Papilionoideae in UniProt (http://www.uniprot.org/), which contained 490,695 sequences when updated on July 6, 2017. The mass tolerance was set to 20 ppm, and the main search tolerance was 6 ppm, allowing for two missed trypsin cleavages. Cysteine carbamidomethylation was set as a fixed modification. Methionine oxidation and N-acetylation protein were set as variable modifications. The false discovery rate (FDR) was set to 0.01 for peptide and protein identification (Additional files 3 and 4). MaxQuant also added a list of common contaminants to the database to avoid false discoveries from contaminant proteins. The experiments contained three biological replicates. Proteins identified at least twice in the three biological replicates were considered quantifiable proteins. Proteins with abundance changes of more than 2-fold and cutoff values of *P* < 0.05 were defined as differentially expressed between the groups (Additional file 5). The Blast2GO (version 3.3.5) program was used for protein mapping and functional annotation against the GO database. The online Kyoto Encyclopedia of Genes and Genomes (KEGG) database (http://www.genome.jp/kegg/) was used to classify and group the identified proteins.

### Quantitative real-time PCR (qRT-PCR) analysis

Total RNA from stylo leaves and roots were extracted using the Trizol reagent (Invitrogen, USA) according to the manual. M-MLV reverse transcriptase (Promega, USA) was used for first strand cDNA synthesis. qRT-PCR analysis was performed using SYBR Premix Ex Taq II (Takara, China), and the reaction was monitored by a Rotor-Gene Q system (Qiagen, Germany). qRT-PCR reaction were set as follows: 95 °C for 1 min, 40 cycles of 95 °C for 15 s, 60 °C for 15 s and 72 °C for 30 s. Fluorescence data were collected at 72 °C. The qRT-PCR primers for the corresponding genes encoding the identified proteins are shown in Additional file 6. Relative gene expression levels were calculated relative to the expression levels of the housekeeping gene *SgEF-1a* [25]. Gene expression analysis contained three biological replicates.

### Statistical analysis

Microsoft Excel 2003 (Microsoft Company, USA) was used to calculate means and standard errors. Data analysis was performed by one-way ANOVA and the Student’s *t* test using the SPSS program (SPSS Institute, USA, version 13.0).

## Additional files

Additional file 1: **Figure S1.** Effects of Mn treatments on the growth of different stylo genotypes. (**a**) SPAD. (**b**) Plant dry weight. After 30 d of normal growth, stylo seedlings were treated with 5 or 400 μM MnSO_4_ for 10 d. Each bar indicates the mean of four biological replicates with standard error. The same letter represents no significant difference at the *P* = 0.05 level. (PDF 167 kb)

Additional file 2: **Table S1.** Number of peptides, proteins and DEPs identified from the samples. (XLSX 9 kb)

Additional file 3: **Table S2.** Information of peptides identified from the leaves and roots of stylo. (XLSX 1836 kb)

Additional file 4: **Table S3.** Information of proteins identified from the leaves and roots of stylo. (XLSX 789 kb)

Additional file 5: **Table S4.** Differentially expressed proteins identified from the leaves and roots of stylo. (XLSX 1133 kb)

Additional file 6: **Table S5.** List of primers used in the study for gene expression analysis. (XLSX 9 kb)

## Abbreviations

14–3-3: 14–3-3 like protein B
A1D: RAB GTPase-like protein A1D
ADH: Alcohol dehydrogenase
AH: Aconitate hydratase
ALS: Acetolactate synthase
AMY: Alpha-amylase
ASD/BXL: Alpha-L-arabinofuranosidase/beta-D-xylosidase
CBSX1: CBS domain-containing protein CBSX1
CD4B: ATP-dependent Clp protease ATP-binding subunit clpA like CD4B
CFI: Chalcone-flavonone isomerase family protein
CHI: Chitinase class Ib
CHS: Chalcone synthase 2
CS: Citrate synthase
ECHI: Endochitinase
EF1a: Elongation factor 1-alpha
EF1g: Elongation factor 1-gamma
eTIF3b: Eukaryotic translation initiation factor 3 subunit B
eTIF3g: Eukaryotic translation initiation factor 3 subunit G
Exp: expansin
FBPA: Fructose-bisphosphate aldolase
FDH: Formate dehydrogenase
FLA: Fasciclin-like arabinogalactan protein 12
FQR1L2: Probable NAD(P)H dehydrogenase (Quinone) FQR1-like 2
G6PI: Glucose-6-phosphate isomerase
GLN: Glutamine synthetase
GLO: Peroxisomal (S)-2-hydroxy-acid oxidase GLO1
GLT: Glutamate synthase
GLU: beta-1,3-glucanase
GluDH: Glutamate dehydrogenase
GRF2: General regulatory factor 2
GST: Probable glutathione S-transferase
H1.2: Histone H1.2
HSP70: Heat shock 70 kDa protein
HSP81–2: Heat shock protein 81–2
HSP90–1: Heat shock protein 90–1
IFR: Isoflavone reductase
IFRL: Isoflavone reductase-like protein
IRK: Putative inactive receptor kinase
L14: 50S ribosomal protein L14
L4: 50S ribosomal protein L4
LAPL1: leucine aminopeptidase 1-like
LHCa/b: Chlorophyll a-b binding protein
LRP: Light-regulated protein
MDH: Cytosolic malate dehydrogenase
ME: Malic enzyme
NQOR: NAD(P)H:quinone oxidoreductase
NUP35: Nuclear pore complex protein NUP35
OCT: Ornithine carbamoyltransferase
OEE: Photosystem II oxygen-evolving enhancer protein
OHK: Osmosensor histidine kinase
Osmotin: Osmotin-like protein
PAL: Phenylalanine ammonia-lyase
PDIL7: Protein disulfide isomerase-like 7
PEPC: Phosphoenolpyruvate carboxylase
PGLP: Phosphoglycolate phosphatase
PNSB2: Photosynthetic NDH subunit of subcomplex B 2
POD: Peroxidase
POP1: Ribonucleases P/MRP protein subunit POP1
Por: NADPH-protochlorophyllide oxidoreductase
PP: Serine/threonine-protein phosphatase
PPO: Polyphenol oxidase
PR10: Pathogenesis-related protein 10
PsaA: Photosystem I P700 chlorophyll a apoprotein A1
PsaC: Photosystem I iron-sulfur center
PsbC: Photosystem II CP43 reaction center protein
QOR: Quinone oxidoreductase-like protein 2 homolog
rbcL: Ribulose bisphosphate carboxylase large chain
RCA: Rubisco activase 2
S17: 30S ribosomal protein S17
S7: 30S ribosomal protein S7
SAMS: S-adenosylmethionine synthase
SDH: Succinate dehydrogenase
SGAT: Serine-glyoxylate aminotransferase
SHMT: Serine hydroxymethyltransferase
STT3B: Dolichyl-diphosphooligosaccharide--protein glycosyltransferase subunit STT3B
SuSy: Sucrose synthase 2
UBC: Ubiquitin-conjugating enzyme
UGGT: UDP-glucose:glycoprotein glucosyltransferase 1
UPL1: E3 ubiquitin-protein ligase UPL1
USP: Universal stress protein
XTH: Xyloglucan endotransglucosylase/hydrolase
α-tubulin: Tubulin alpha chain
β-tubulin: Tubulin beta-1 chain
