# Supplementary material for: Physiological responses and proteomic changes reveal insights into Stylosanthes response to manganese toxicity
Source: BMC Plant Biol. 2019 May 22;19:212. doi: 10.1186/s12870-019-1822-y (PMC6530018; doi:10.1186/s12870-019-1822-y)
Supplement: Supplementary file 1 — Figure S1. Effects of Mn treatments on the growth of different stylo genotypes. (a) SPAD. (b) Plant dry weight. After 30 d of normal growth, stylo seedlings were treated with 5 or 400 μM MnSO4 for 10 d. Each bar indicates the mean of four biological replicates with standard error. The same letter represents no significant difference at the P = 0.05 level. (PDF 167 kb) [file 12870_2019_1822_MOESM1_ESM.pdf]

Fig. S1

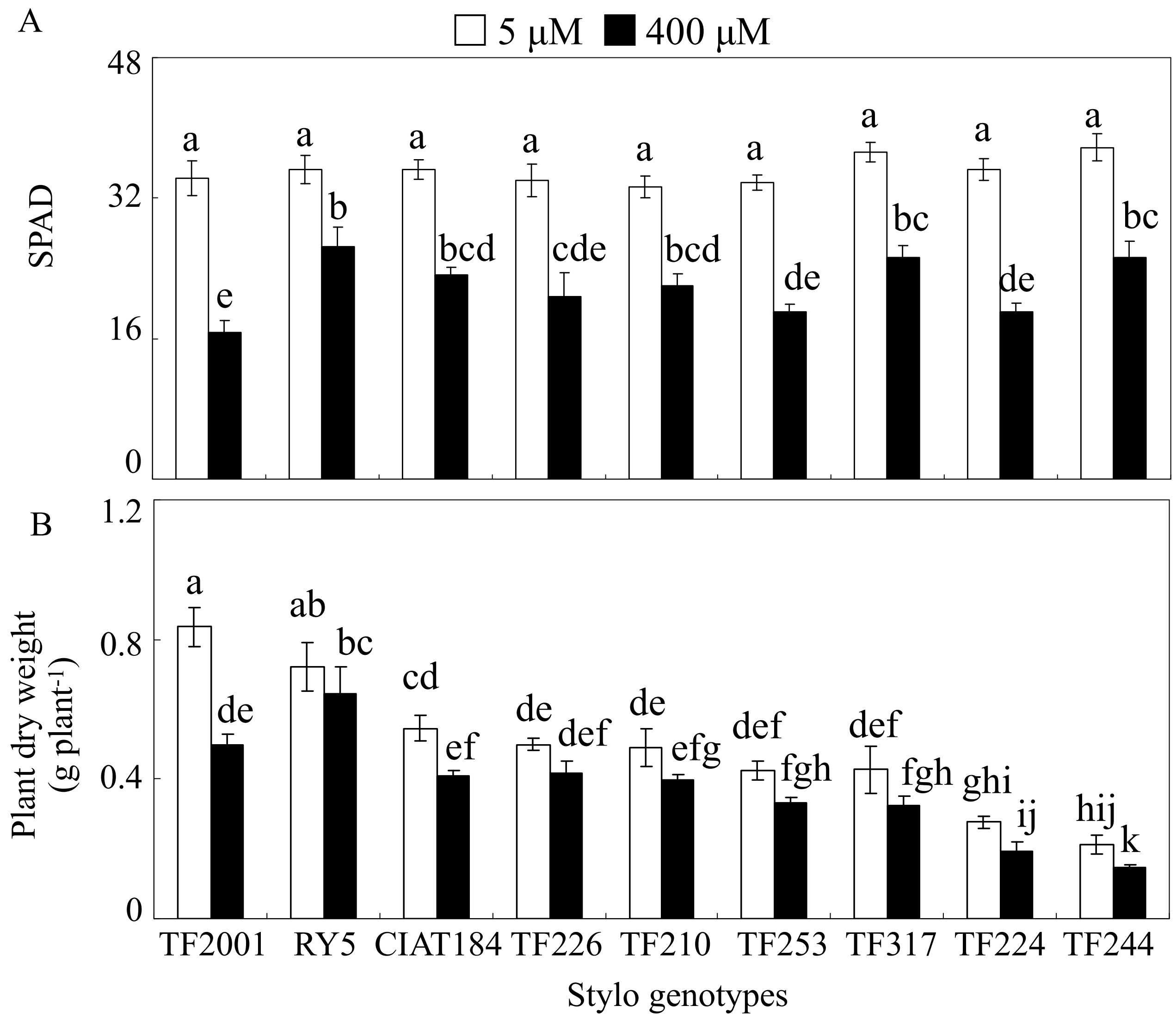

Additional file Fig. S1. Effects of Mn treatments on the growth of different stylo genotypes. (a) SPAD. (b) Plant dry weight. After 30 d of normal growth, stylo seedlings were treated with 5 or 400 μM $MnSO_4$ for 10 d. Each bar indicates the mean of four biological replicates with standard error. The same letter represents no significant difference at the *P*=0.05 level.
